# *Ziziphus jujube* promotes fertility and pregnancy outcomes in Rat model of uterine adhesions

**DOI:** 10.3389/fphar.2024.1496136

**Published:** 2025-01-27

**Authors:** Fereshteh Asgharzadeh, Mahsa Attarian, Majid Khazaei, Abdulridha Mohammed Al-Asady, Saeide Mansoori, Hamideh Naimi, Moein Eskandari, Azar Khorrami, Seyedeh Elnaz Nazari, Akram Aminian, Marjaneh Farazastanian, Elaheh Eshtad, Amir Avan, Mikhail Ryzhikov, Malihe Hasanzadeh, Seyed Mahdi Hassanian

**Affiliations:** ^1^ Metabolic Syndrome Research Center, Mashhad University of Medical Sciences, Mashhad, Iran; ^2^ Department of Medical Physiology, Faculty of Medicine, Mashhad University of Medical Sciences, Mashhad, Iran; ^3^ Department of Clinical Biochemistry, Faculty of Medicine, Mashhad University of Medical Sciences, Mashhad, Iran; ^4^ Department of Medical Sciences, Faculty of Nursing, Warith Al-Anbiyaa University, Kerbala, Iraq; ^5^ Department of Medical Sciences, Faculty of Dentistry, University of Kerbala, Kerbala, Iraq; ^6^ Department of Pharmacology, Faculty of Medicine, Mashhad University of Medical Sciences, Mashhad, Iran; ^7^ Medical School, Mashhad University of Medical Sciences, Mashhad, Iran; ^8^ Department of Physiology and Pharmacology, Afzalipour Faculty of Medicine, Kerman University of Medical Sciences, Kerman, Iran; ^9^ Department of Obstetrics and Gynecology, Faculty of Medicine, Mashhad University of Medical Sciences, Mashhad, Iran; ^10^ Department of Human Genetics, Faculty of Medicine, Mashhad University of Medical Sciences, Mashhad, Iran; ^11^ School of Medicine, Saint Louis University, Saint Louis, MO, United States

**Keywords:** *Ziziphus jujube*, uterine adhesion, pregnancy outcomes, endometrial regeneration, Asherman’s syndrome, embryonic development

## Abstract

**Introduction:**

The therapeutic efficacy of oral administration of *Ziziphus jujube* in the context of uterine adhesion (UA) and its impact on pregnancy outcomes was investigated.

**Methods:**

In a rat UA model, *Z. jujube* was evaluated for its ability to mitigate injury-induced uterine adhesion bands, uterine shortening, and enhance endometrial regeneration. The assessment included analysis of gland numbers, uterine endometrial thickness, and regulation of inflammatory cytokines. The antioxidant properties of *Z. jujube* were also studied through antioxidant enzyme activity in uterine tissue homogenates. Fibrotic changes were examined through histological Trichrome staining and analysis of pro-fibrotic factors.

**Results:**

Treatment with *Z. jujube* resulted in a significant reduction in uterine tissue fibrosis, as evidenced by histological evaluation and reduced expression of fibrotic markers. The intervention demonstrated positive outcomes in embryonic development, pregnancy rates, and pregnancy outcomes. *Z. jujube* effectively inhibited the formation of extra-uterine adhesion bands to internal organs. No toxicity-related morphological changes were observed in vital organs of the *Z. Jujube*-treated group.

**Discussion:**

The results collectively indicate that *Z. jujube* is a safe and potent natural product with anti-inflammatory and anti-fibrotic properties, highlighting its potential as a novel candidate for clinical studies targeting UA in patients.

## Introduction

Asherman’s syndrome is a worldwide disease affecting women of reproductive age (Deans and Abbott, 2010). Typically, when the basal layer of the endometrium undergoes mechanical or infectious damage, it sets off an inflammatory response that triggers the formation of fibrous adhesion bands within the uterine cavity. This disruption of the endometrial regeneration process results in an alteration of the normal biological activity of the endometrium (Evans-Hoeker and Young, 2014; Chen et al., 2021). IUA is a leading cause of secondary infertility in women, affecting approximately 25%–30% of individuals struggling with infertility (Rein et al., 2011; Zhang et al., 2019). Moreover, menstrual irregularities, notably amenorrhea, and decreased pregnancy rates with poor outcomes are significant complications associated with IUA (Deans and Abbott, 2010; Al-Inany, 2001). Today, various therapeutic approaches are employed worldwide to inhibit the fibrosis process and the subsequent formation of adhesion bands. These approaches include hysteroscopic adhesiolysis, a surgical procedure for removing visible adhesion bands, the use of intrauterine devices (IUDs), intrauterine gel injections, and estrogen therapy aimed at preventing adhesion recurrence and stimulating endometrial regeneration (Zhang et al., 2019; Myers and Hurst, 2012; Tsui et al., 2014). Despite demonstrating effectiveness in treating IUA, these methods are somewhat invasive, outcomes can vary among individuals, and they are not universally accessible. Therefore, the development and proposal of new methods with greater effectiveness in endometrial regeneration and lower invasiveness are warranted (Yu et al., 2008; Dreisler and Kjer, 2019).

Inflammation and its successive fibrosis are the principal players in the adhesion formation process (Wang et al., 2017; Leung et al., 2021; Xue et al., 2015). Following an injury to the endometrium, inflammatory signaling pathways become activated, leading to an imbalance in the oxidant/antioxidant ratio and increased secretion of inflammatory mediators, including IL-6, IL-1β, IFN-γ, TNF-α, and the key regulator of endometrial fibrosis, TGF-β (Wang et al., 2017; Wyble et al., 1997; Tak and Firestein, 2001; Freudlsperger et al., 2013; Jones et al., 2006). Once TGF-β is activated, it stimulates collagen deposition, increases fibrosis percentage, and enhances endometrial stiffness, ultimately resulting in the formation of uterine adhesion bands (Leung et al., 2021; Liu and Wang, 2022).

The fruit of *Ziziphus jujube* (*Z*. *jujube*) is a traditional medicinal herb (Plastina et al., 2012; Chen and Tsim, 2020; Huang et al., 2007) with known anti-inflammatory and fibrinolytic properties, containing high concentrations of vitamins C and B, as well as various minerals such as magnesium, phosphorus, potassium, sodium, and zinc (Li et al., 2007). The major active components in *Z*. *jujube* responsible for its bioactivities include triterpenic acids, flavonoids, polysaccharides, and saponins. These bioactive compounds exhibit various pharmacological properties such as antioxidant, anti-inflammatory, and immunomodulatory effects. In terms of absorption, studies suggest that these components can be effectively absorbed due to their optimal molecular size and the presence of transporters in the body that facilitate their uptake. Research on the bioavailability and pharmacokinetics of these active components in *Z*. *jujube* is ongoing to further understand their absorption mechanisms and potential health benefits (Hong et al., 2020; Gao et al., 2013). Numerous studies have demonstrated the potent ability of either the fruit or extract of *Z. jujube* to attenuate inflammation (Zhan et al., 2018; Mesaik et al., 2018; Shen et al., 2022; Ruan et al., 2021; Chen et al., 2014; Liu et al., 2020; Farhadnejad et al., 2022), oxidative stress (Khajavi Rad et al., 2021; Wang et al., 2012), and fibrosis (Wang et al., 2012) in cellular, animal, and clinical studies.

In our previous studies, we explored the therapeutic potential of various components against adhesion band formation in different organs, including tendons (Askarnia-Faal et al., 2023), the abdomen (Arjmand et al., 2020; Soleimani et al., 2020), among others. This study uniquely investigates the therapeutic potential of *Z. jujube*, a natural product with anti-inflammatory and anti-fibrotic properties, in addressing uterine adhesions, which has not been extensively explored in prior studies. By highlighting the innovative approach of utilizing *Z. jujube* to improve fertility outcomes and endometrial regeneration, this research contributes valuable insights that may pave the way for new, effective treatment strategies for women suffering from Asherman’s syndrome, thereby filling a critical gap in the current therapeutic landscape.

## Materials and methods

### Materials

Jujube was purchased locally and powdered using a blender. Rat TNF-α, IL-6 and TGF-β ELISA kits were obtained from Zellbio (Lonsee, Germany). Malondialdehyde (MDA), Superoxide Dismutase (SOD), and Catalase (CAT) assay reagents were acquired from Kushan Zist Azma Co. (Tehran, Iran). All other reagents were purchased from Sigma-Aldrich (St. Louis, MO, United States).

### Study design

Female albino Wistar rats aged 8 weeks were purchased from the laboratory animal center of Mashhad University of Medical Sciences, Mashhad, Iran. The present study was conducted in compliance with the ARRIVE guidelines and guidelines of the Research Ethics Committee of Mashhad University of Medical Sciences with the ID number IR. MUMS.AEC.1401.084.

The model induction involved mechanically injuring the uterus during the estrus cycle. Vaginal smearing was performed daily at 8 a.m. to assess the estrus cycle of rats. The induction procedure included anesthesia via intra-peritoneal injection of a ketamine (80 mg/kg)/xylazine (8 mg/kg) mixture. Subsequently, midline incisions were made to access the uterus, and the endometrium layer of both uteri was gently scratched using a 7-gauge needle (Liang et al., 2022). The incision area was then sutured. A total of 72 female rats were divided into three groups (n = 24 for each group): Sham Group (midline excision without uterine damage), IUA Positive Control Group (uterine damage with no treatment), and *Z. jujube*-Treated Group (uterine damage followed by 10 days of 400 mg/kg/day *Z. jujube* oral treatment). In our previous study (Khalili-Tanha SEN et al., 2024), we employed 400 mg/kg/day *Z. jujube* and observed notable anti-inflammatory and anti-fibrotic effects, along with a reduction in adhesive bands in the abdomen. The positive outcomes obtained from our earlier research further solidified our decision to utilize the same dosage in the current study. However, considering the evidence from the existing literature (Resim et al., 2020; Wang et al., 2012) and our research, we believe that the 400 mg/kg/day *Z. jujube* was the most appropriate choice for our study.

### Three phases of the study

Phase 1: Ten days post-surgery, 24 rats (8 per group) were sacrificed to investigate the stimulatory effect of *Z. jujube* on endometrial layer regeneration. Molecular and histological examinations of uterine tissues were conducted to evaluate inflammatory and fibrotic markers as well as regeneration indicators. Extra-uterine adhesions to other abdominal sites and adjacent organs were assessed using an adhesion scoring system (Güney et al., 2017).

Phase 2: On the 10th day post-injury, 24 rats (8 per group) were housed with male rats for mating. After 15 days post-mating, pregnant rats were sacrificed, and parameters such as the number, weight, implantation of embryos, and placental weight were compared between groups. Non-pregnant rats were subsequently housed with male rats to induce further pregnancies.

**FIGURE 1:**
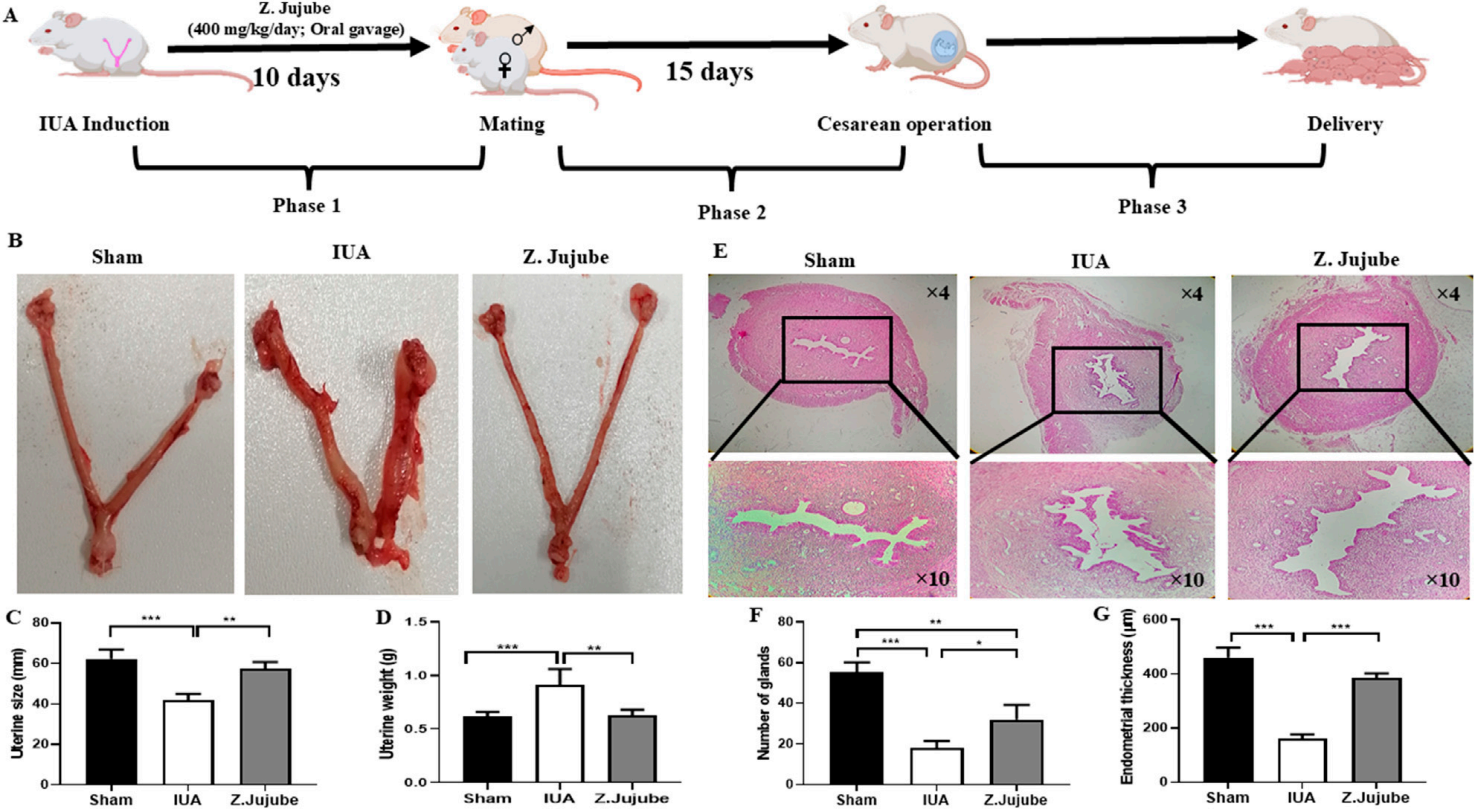
*Ziziphus jujube* decreased adhesion bands and enhanced endometrial regeneration. **(A)** The schematic presentation of the study. The therapeutic effects of *Ziziphus jujube* on **(B, C)** uterine size and **(D)** weight were investigated in different groups. **(E)** H&E-stained sections of the rats’ uterine. **(F)** The number of glands and **(G)** endometrial thickness were also measured in the H&E-stained sections of the uterine. *P < 0.05, **P < 0.01, ***P < 0.001. Data were presented as Mean ± SEM.

Phase 3: This phase evaluated the phenotypic and neonatal outcomes of the pregnancy. Twenty-four rats (8 per group) were housed with male rats for mating. Similar to Phase 2, animals were maintained under normal housing conditions until birth. Outcomes included the number and weight of babies, the number of live births, conception time, and a 2-month evaluation of the growth rate of live babies. A schematic representation of the study design is provided in Figure 1. After the experiment, euthanasia was conducted via carbon dioxide (CO_2_) inhalation.

### Histological evaluation

The histological assessment involved Hematoxylin and Eosin (H&E) staining to examine endometrial alterations and morphology as described (Feldman and Wolfe, 2014). Measurements of endometrial thickness and the number of glands were performed. Trichrome staining was utilized to evaluate the extent of fibrosis in uterine samples as described (Sridharan et al., 2022) and fibrotic areas were quantified using ImageJ software (NIH, Maryland, United States).

### Quantitative RT-PCR

Real-time quantitative PCR (qRT-PCR) was performed as previously described (Hassanian et al., 2014). Total RNA was extracted from liquid nitrogen-frozen tissues, followed by cDNA synthesis via kit (Yekta Tajhiz, Tehran, Iran). qRT-PCR amplification was carried out using Ampliqon SYBR Green PCR Master Mix, with GAPDH serving as the control housekeeping gene. Primer sequences are presented in Table 1.

**TABLE 1:** The primers used for qRT-PCR.

Gene	Source	Primer	Sequence
GAPDH	Rat	Forward	5′-CTT​CTC​TTG​TGA​CAA​AGT​GGA​CA-3′
		Reverse	5′-TTG​ACT​GTG​CCG​TTG​AAC​TTG-3′
IL-1β	Rat	Forward	5′-GAC​TTC​ACC​ATG​GAA​CCC​GT-3′
		Reverse	5′-GGA​GAC​TGC​CCA​TTC​TCG​AC-3′
IFN-γ	Rat	Forward	5′-TGA​GCA​TCG​CCA​AGT​TCG​AG-3′
		Reverse	5′-TCT​GGT​GAC​AGC​TGG​TGA​ATC-3′
TNF-α	Rat	Forward	5′-AGG​CTG​TCG​CTA​CAT​CAC​TG-3′
		Reverse	5′-CTC​TCA​ATG​ACC​CGT​AGG​GC-3′
α-SMA	Rat	Forward	5′-GGC​TAT​TCC​TTC​GTG​ACT​ACT​G-3′
		Reverse	5′-CAG​TGG​CCA​TCT​CAT​TTT​CAA​AG-3′

### ELISA assay

The tissue concentrations of TNF-α, IL-6, and TGF-β were measured using Zellbio ELISA kits according to the manufacturer’s protocol.

### Oxidative stress evaluation

Oxidative stress markers, including MDA as an oxidative marker, and the enzyme activities of SOD and CAT as antioxidant markers, were measured in uterine tissue homogenates as previously described (Hashemzehi et al., 2020).

### Scoring of extra-uterine adhesions

At the end of Phase 1, two independent observers, blinded to the groups, scored pelvic adhesions from sacrificed animals according to the adhesion scoring system developed by Mazuji et al. (1964) (Tables 2–4).

**TABLE 2:** The extent score of adhesion formation.

Grade	Description
0	No uterine adhesion
1	1%–25% involvement
2	26%–50%
3	51%–75%
4	76%–100%

**TABLE 3:** The severity score of adhesion formation.

Grade	Description
0	No adhesion
1	Filmy avascular
2	Vascular or opaque
3	Cohesive attachment of uterine horn to each other or other abdominal organs

**TABLE 4:** The degree score of adhesion formation.

Grade	Description
0	No adhesion
1	The adhesion could be separated from tissue with gentle traction
2	The adhesion could be separated from tissue with moderate traction
3	Requiring sharp dissection

### Statistical analysis

The data obtain from groups were statistically compared using one-way ANOVA followed by LSD *post hoc* test. All the quantified numeral values are presented as mean ± SEM. P values < 0.05 indicated a significant difference between the given groups. *p < 0.05, **p < 0.01, ***p < 0.001.

## Results

### *Z. jujube* decreased adhesion band formation and enhanced endometrial regeneration in rat IUA model

This study is comprised of three phases (Figure 1A). At the end of the first phase, we evaluated the macroscopic and microscopic effects of *Z. jujube* treatment on the formation of adhesion bands and endometrial regeneration. Our results showed that oral administration of *Z. jujube* significantly decreased uterine shortening (Figures 1B, C) and decreased uterine weight (Figure 1D) compared to the untreated IUA group. Uterine shortening was associated with fibrosis, while uterine weight was associated with inflammation (Kim et al., 2020). Additionally, H&E-stained sections of the uterine revealed enhanced endometrial regeneration, visualized by a significant increase in the number of glands (Figure 1E) and endometrial thickness (Figure 1F) in *Z. jujube*-treated rats.

### *Z. jujube* attenuated uterine inflammation in rat IUA model

**FIGURE 2:**
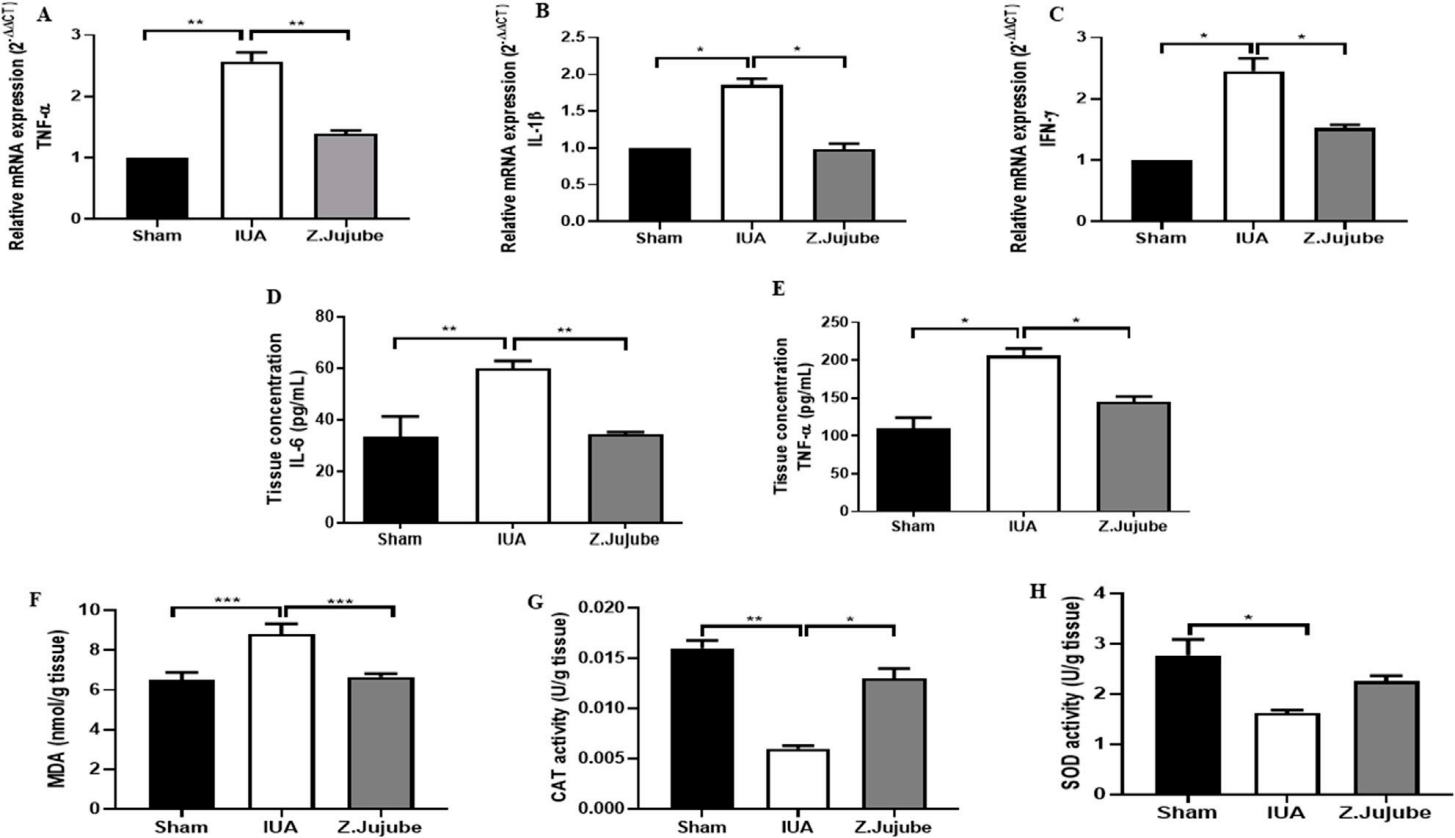
*Z. jujube* attenuated uterine inflammation in rat IUA model. The transcriptional mRNA level of **(A)** TNF-α, **(B)** IL-1β, and **(C)** IFN-γ were measured in the uterine tissue samples of the animals. Protein expression level of **(D)** IL-6 and **(E)** TNF-α was also evaluated in the tissue homogenates of the uterine samples. **(F)** *Z. jujube* attenuated MDA concentration, whereas **(G)** increased enzymatic activities of CAT, and **(H)** SOD, in uterine tissue samples. *P < 0.05, **P < 0.01, ***P < 0.001. Data were presented as Mean ± SEM.

To investigate the protective mechanism of *Z. jujube*, we compared the expression levels of pro-inflammatory molecules including IL-1β, IL-6, IFN-γ, and TNF-α in the uterine tissue samples. Our results showed that *Z. jujube* potently downregulated mRNA expression of TNF-α (Figure 2A), IL-1β (Figure 2B), and IFN-γ (Figure 2C) compared to the un-treated IUA group. Furthermore, protein concentrations of IL-6 (Figure 2D) and TNF-α (Figure 2E) were significantly decreased in the *Z. jujube*-treated group. To further determine the anti-inflammatory properties of *Z. jujube*, concentrations and the enzymatic activities of oxidant/antioxidant markers were evaluated in uterine tissue homogenates. *Z. jujube* reduced MDA concentrations (Figure 2F), an oxidant marker, while increasing the enzymatic activities of CAT (Figure 2G) and SOD (Figure 2H), two antioxidant markers, in uterine tissue.

### *Z. jujube* elicited fibrinolytic effects in the uterine tissues

**FIGURE 3:**
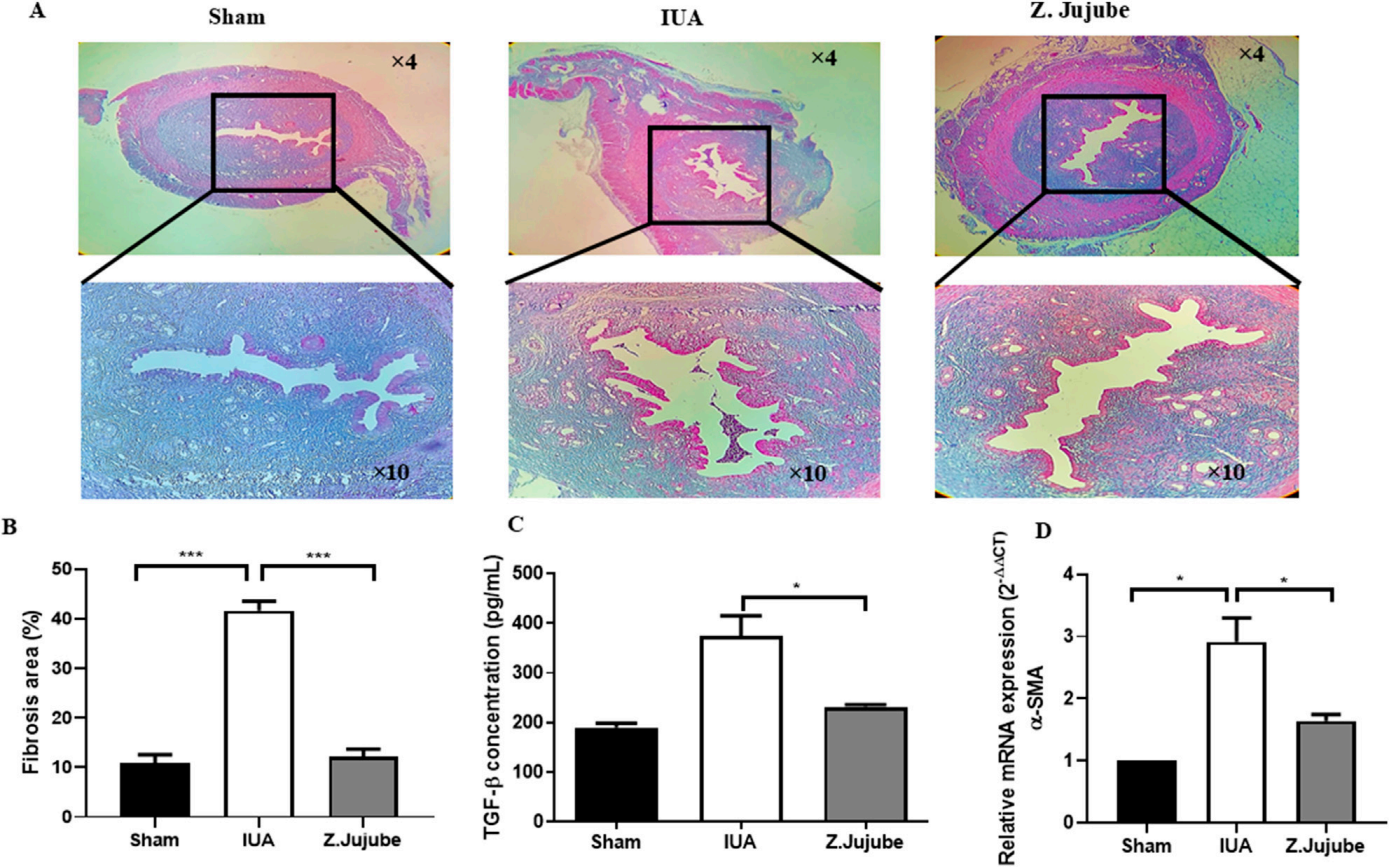
*Z. jujube* elicited anti-fibrotic effects in the uterine tissues. **(A)** *Z. jujube* decreased collagen deposition in the uterine tissue sections as visualized by Trichrome staining. **(B)** Fibrosis percentage was compared and quantified in the uterine histological sections of rats. *Z. jujube* decreased **(C)** protein concentrations of TGF-β and **(D)** mRNA level of α-SMA in the uterine tissue samples. *P < 0.05, **P < 0.01, ***P < 0.001. Data were presented as Mean ± SEM.

Fibrosis is a key factor in the pathogenesis of adhesion band formation in the uterus. The anti-fibrotic properties of *Z. jujube* were investigated in uterine tissue samples of animals. Results showed that *Z. Jujube* potently decreased collagen deposition in the uterine tissue sections as visualized by trichrome staining (Figure 3A). Similarly, the percentage of fibrosis was decreased in the uterine histological sections of rats orally treated with *Z. jujube* compared with the untreated IUA group (Figure 3B). Transforming Growth Factor-β (TGF-β) is a pivotal player in tissue fibrosis (Verrecchia and Mauviel, 2007). Our results showed that *Z. Jujube* downregulated the expression of TGF-β (Figure 3C) and pro-fibrotic genes such as α-SMA (Figure 3D) in uterine tissue samples.

### Effect of *Z. jujube* on the embryonic development in the rat IUA model

**FIGURE 4:**
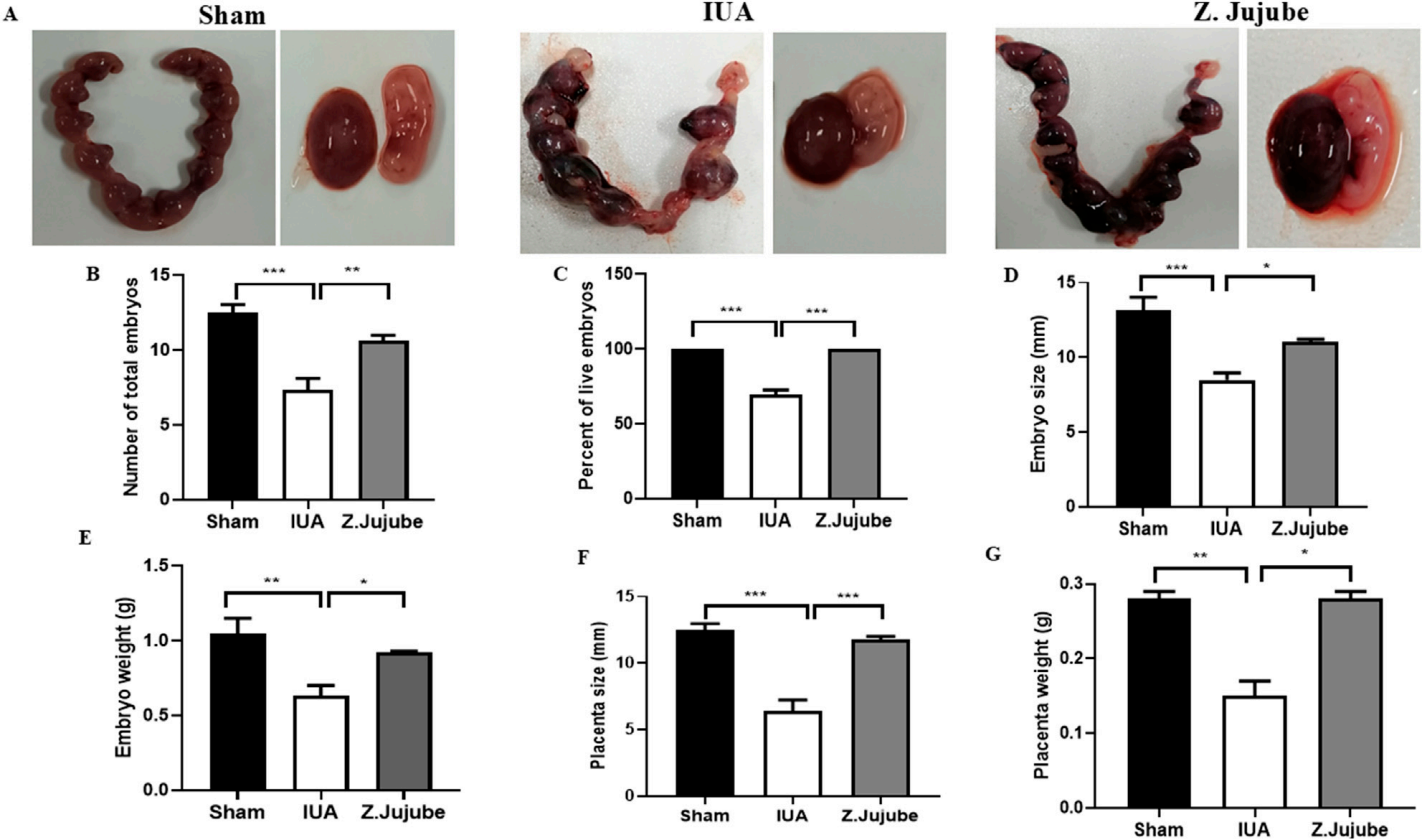
*Z. jujube* enhanced embryonic development in the rat model of IUA. **(A)** Macroscopic comparison of uterine, embryo and placenta in different groups of the study. Gavage administration of *Z. jujube* post-injury enhanced embryonic development as indicated by an increase in **(B)** number of total embryos, **(C)** the percent of live embryos, **(D)** embryo size, **(E)** embryo weight, **(F)** placenta size and **(G)** placenta weight. *P < 0.05, **P < 0.01, ***P < 0.001. Data were presented as Mean ± SEM.

In the second phase of the study, the effects of *Z. jujube* on factors related to embryonic development were assessed in the uterine of pregnant rats. Our results showed that injury to the un-treated uterine IUA group decreased the total number of embryos (Figure 4A), while oral administration of *Z. jujube* post-injury increased the number (Figure 4B), percent of live embryos (Figure 4C), size (Figure 4D), and weight of embryos (Figure 4E). Moreover, injury-induced decrease in fetus implantation factors, including placenta size and weight in the untreated group, were significantly improved in the presence of *Z. jujube* treatment (Figures 4F, G), demonstrating potent protective effects of gavage administration of *Z. jujube* on embryonic development in the rat uterus post-injury.

### *Z. jujube* enhanced the pregnancy outcomes in a rat model of IUA

**FIGURE 5:**
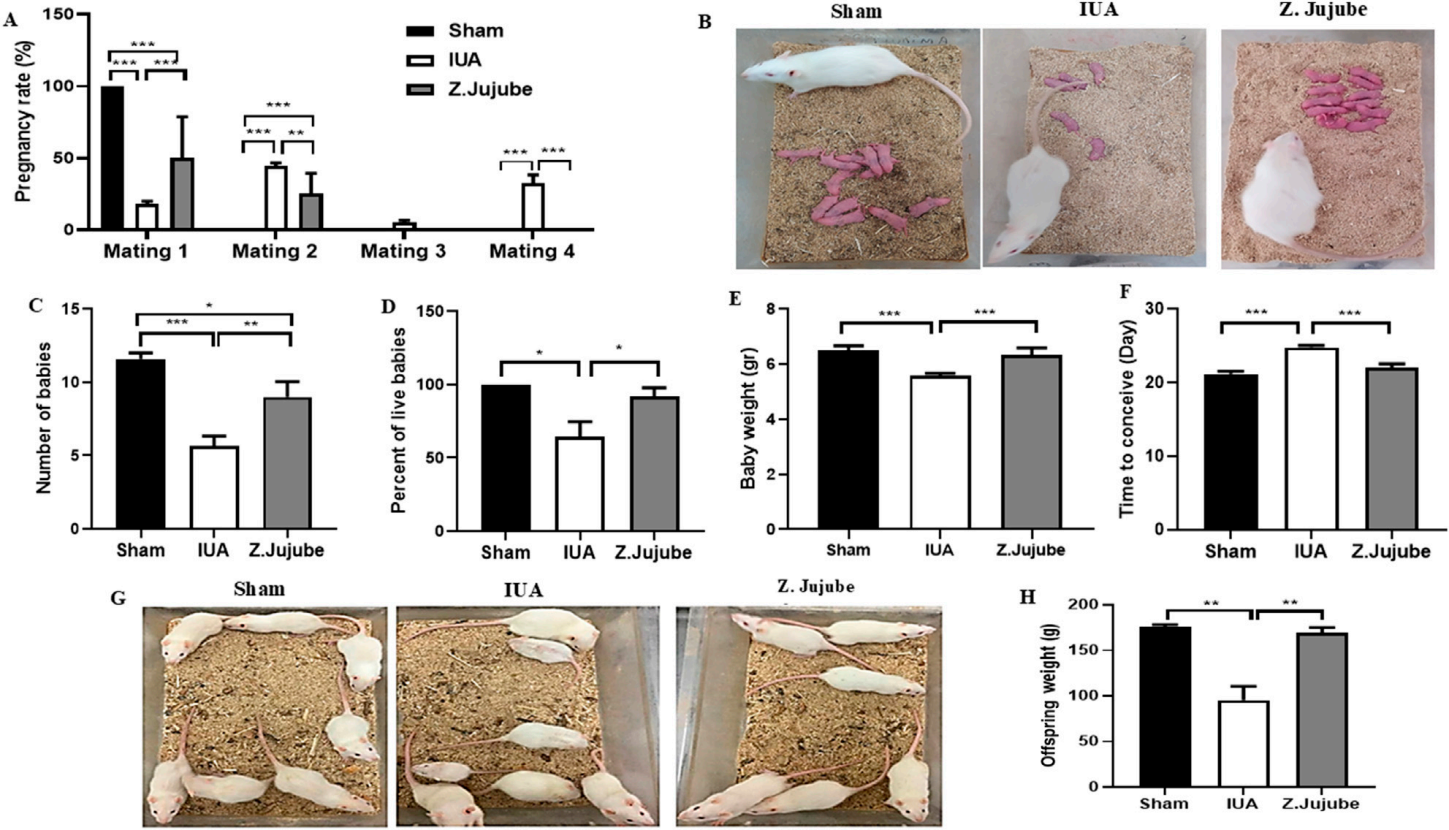
Pregnancy outcomes were enhanced by *Z. jujube* treatment. **(A)** The rate of pregnancy is compared between groups. *Z. jujube* treatment improved the pregnancy outcomes as **(B–C)** the number of babies, **(D)** the percent of live babies, and **(E)** the weight of babies increased in the jujube-treated rats. **(F)** Mean conception time of each given group. **(G)** Babies of *Z. jujube*-treated rats showed normal growth patterns into adulthood and **(H)** were significantly heavier than the ones in the IUA group.*P < 0.05, **P < 0.01, ***P < 0.001. Data were presented as Mean ± SEM.

In the third phase of the study, we compared the pregnancy outcomes between groups. Injury-induced adhesion formation in the IUA group resulted in a significant reduction in the pregnancy rate in rats. However, this reduction was abrogated by *Z. jujube* treatment, as all the animals in the *Z. jujube*-treated group had successful pregnancies by the second mating attempt, in contrast to the IUA group, where about 40% of the rats got pregnant at the fourth mating attempt (Figure 5A). The number of babies (Figures 5B, C), and the percentage of live babies (Figure 5D) were significantly increased by *Z. jujube* treatment and the babies were also heavier than in the IUA group (Figure 5E). Furthermore, *Z. jujube*-treated rats conceived significantly faster, in fewer days, compared to the IUA control rats (Figure 5F).

To further evaluate the effect of IUA on the normal growth of the animals, we maintained the babies in the standard condition for 2 months. Interestingly, we observed impaired growth patterns in some of the babies in the IUA control group, as indicated by a wide variation in their sizes (Figure 5G). On the other hand, the babies of the *Z. jujube*-treated rats showed a more balanced and normal growth pattern. Consistently, the weight of the babies in the *Z. jujube*-treated group was the same as the Sham group and significantly higher than that of the IUA rats (Figure 5H).

### *Z. jujube* suppressed extra-uterine adhesion band formation to the internal organs

**FIGURE 6:**
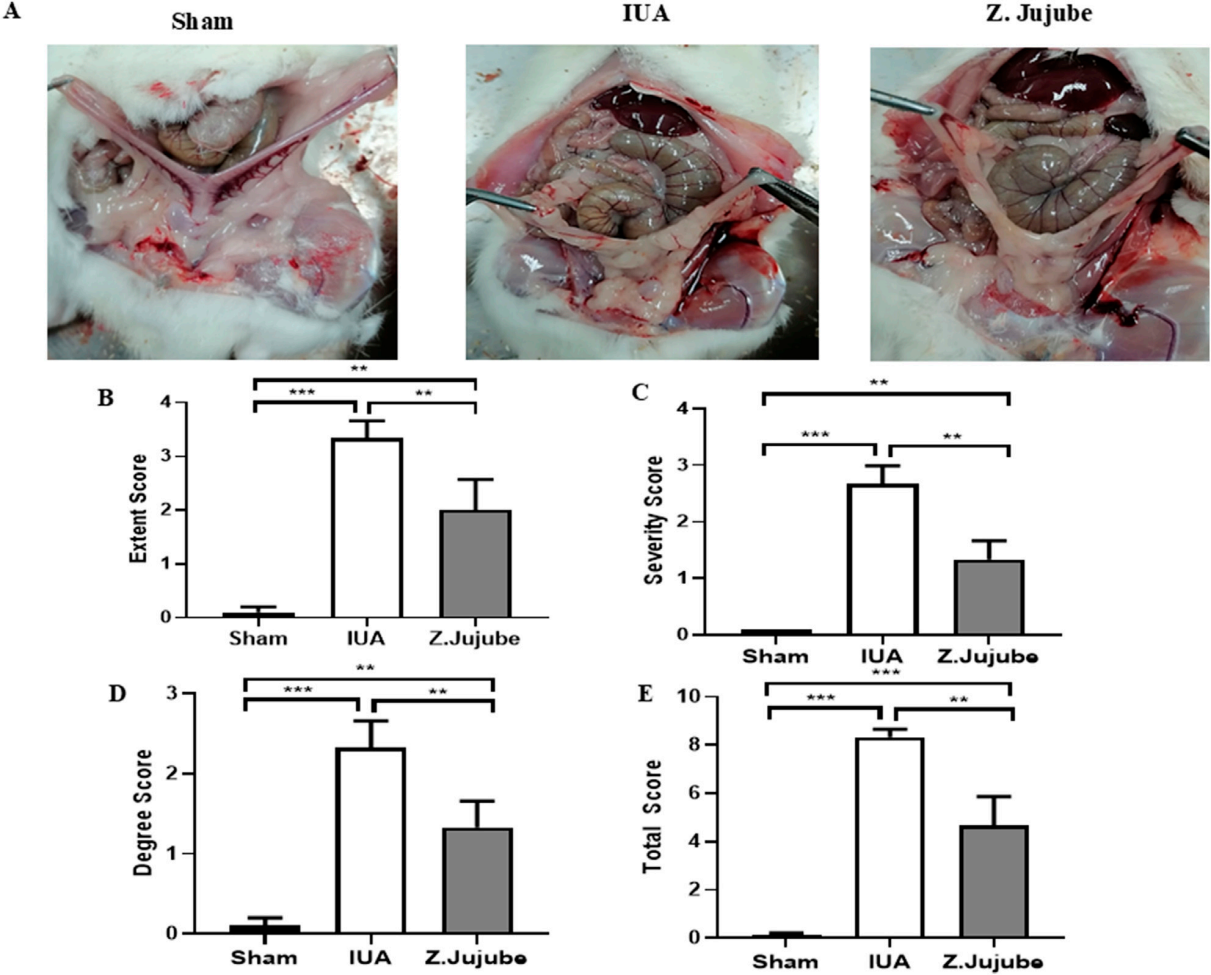
*Z. jujube* decreased extra-uterine adhesions. **(A)** Compared to the IUA group, adhesion of uterine to internal organs were decreased in the *Z. jujube*-treated group. **(B)** The extent, **(C)** severity, **(D)** degree, and **(E)** Total score of extra-uterine adhesions were compared between different groups. *P < 0.05, **P < 0.01, ***P < 0.001. Data were presented as Mean ± SEM.

Surgical procedures on the uterus, including cesarean sections, induce the formation of adhesion bands between the uterus and internal organs (Awonuga et al., 2011). Induction of IUA requires the midline excisions of the uterus, which indirectly causes extra-uterine adhesion as well. Therefore, we evaluated the therapeutic potency of *Z. jujube* in decreasing extra-uterine adhesion to internal organs using Mazuji et al. (1964) scoring system. As presented in Figure 6A, oral administration of *Z. jujube* decreased the formation of extra-uterine adhesion to internal organs. Furthermore, using Mazuji et al. scoring system, the adhesion extent score (Figure 6B) (Table 2), severity score (Figure 6C) (Table 3), degree score (Figure 6D) (Table 4), and total adhesion score (Figure 6E) were all attenuated in the *Z. jujube*-treated rats, compared with the untreated IUA group.

### *Z*. *jujube* did not exhibit any histopathological changes in the organs of rats

**FIGURE 7:**
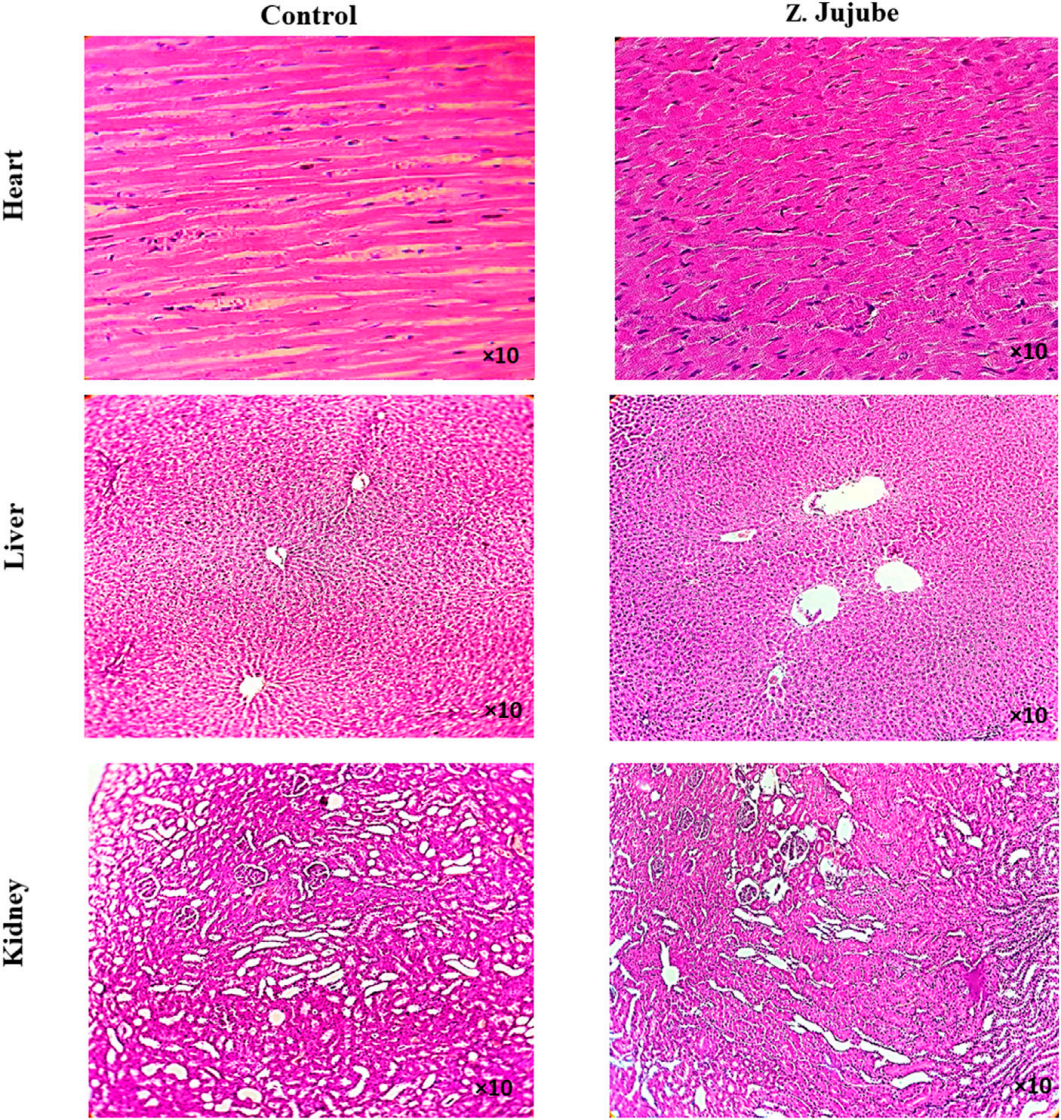
Oral administrations of *Z. jujube* is safe to internal organs. The H&E staining of heart, liver, and kidney tissues showed no histo-pathological changes, infiltration of inflammatory cells to liver or kidney, nor re-arrangement of myofibers in heart in *Z. jujube*-treated groups.

To investigate the potential side effects of *Z. jujube* on treated rats, the heart, kidney, and liver tissues were collected and evaluated by H&E staining. No toxicity-associated morphological changes, no infiltration of inflammatory cells to the liver or kidney, nor re-arrangement of myofibers in the heart were observed in *Z. jujube*-treated groups (Figure 7), supporting the safety of the oral administration of *Z. jujube* on the treated group.

## Discussion

This is the first study investigating the therapeutic potential of *Z. jujube* in a rat model of intra- and extra-uterine adhesion, shedding light on its promising effects. Our results demonstrate that oral administration of *Z. jujube* exerts a significant mitigating influence on the formation and severity of adhesion bands. This effect is demonstrated by the suppression of inflammatory and fibrotic factors, a conclusion drawn from both molecular and histological assessments. Notably, the administration of *Z. jujube* also leads to improvements in pregnancy outcomes, encompassing increased pregnancy and implantation rates, reduced time to conception, and enhanced parameters related to pregnancy, such as the number and weight of offspring in the rat model of IUA.

Intrauterine adhesion (IUA) frequently results from mechanical or infectious injury to the endometrium layer of the uterus, initiating a pro-inflammatory response marked by the expression and release of pro-inflammatory mediators, including TNF-α, IL-6, and IFN-γ (Chen et al., 2018). The anti-inflammatory effects of *Z. jujube* have been investigated in several cellular and animal studies. In line with this, Shen et al. (2022) showed that the immunomodulatory properties of *Z. jujube* polyphenols reduced LPS-induced inflammation by inhibition of NF-κB and MAPK signaling and the reduced production of pro-inflammatory cytokines TNF-α, IL-6, and IL-1β. *Z. jujube* triterpenes were also reported to exert anti-inflammatory effects by ameliorating the production of IL-6 and TNF-α in LPS-stimulated RAW264.7 cells (Ruan et al., 2021). Jujube polysaccharides also ameliorated colitis-related clinical symptoms and histological alterations in C57BL/6 mice by inhibiting NF-κB and MAPK inflammatory signaling pathways (Liu et al., 2020). The *Z. jujube* extract was found to decrease the expression of IL-6 and IL-1β in murine macrophages induced by LPS (Chen et al., 2014). In line with these studies, we showed that oral *Z. jujube* inhibited inflammatory response in the uterine tissue of animals as indicated by downregulated tissue expression of key inflammatory markers including TNF-α, IL-β, and IL-6. Moreover, the tissue concentration of IL-6 and TNF-α -proteins was reduced in *Z. jujube*-treated animals.

The major inflammatory cells in uterine adhesions are typically macrophages and neutrophils. Macrophages play a crucial role in the inflammatory response and tissue repair processes, while neutrophils are involved in the initial stages of inflammation (Zhu et al., 2023).

Regarding the effect of jujube on these immune cells, studies have shown that certain bioactive compounds present in *Ziziphus jujube*, such as flavonoids and triterpenoids, have anti-inflammatory properties. These compounds can modulate the activity of inflammatory cells and cytokines, thereby reducing the inflammatory response in various tissues, including the uterus. While specific research on the direct effect of jujube on inflammatory cells in uterine adhesions is limited, the anti-inflammatory properties of jujube suggest potential benefits in mitigating inflammation and immune cell activation in this context (Batovska et al., 2024; Ruan et al., 2023).

Consistent with the anti-inflammatory properties of *Z. jujube*, its inhibitory effects on oxidative stress have been investigated in previous studies. For instance, Resim et al. showed that *Z. jujube* induced the activity of SOD and CAT, reducing MDA levels of cavernous nerve injury (CNI)-induced erectile dysfunction (ED) in a rat model (Wang et al., 2012). Similarly, *Z. jujube* was reported to exert anti-oxidant properties in a rat model of adriamycin-induced organ toxicity (Khajavi Rad et al., 2021). In another study conducted by Hong et al., *Z. jujube* effectively decreased the oxidative stress in a model of alcohol-induced liver damage, which was indicated by a significant reduction in MDA level (Hong et al., 2020). Using an animal model of diabetes, Hemmati et al. (2015) showed an increase in total antioxidant capacity in the sera of animals accompanied by a reduction in MDA level following *Z. jujube* treatment. Our results showed that *Z. jujube* decreased oxidative stress and regulated the oxidant/antioxidant balance by stimulating the antioxidant markers including SOD and CAT enzymes and inhibiting the oxidative stress marker, MDA.

During the inflammatory phase, immune cells rapidly release pro-inflammatory cytokines to the uterine cavity leading to initiation of fibrotic responses (Leung et al., 2021). Inflammation-induced TGF-β activation stimulates the release of collagens, smooth muscle actin, and other extracellular matrix (ECM) components. Excessive release of ECM contents forms adhesion bands within the uterine cavity (Chen et al., 2021; Leung et al., 2021). *Z. jujube* has been previously reported to suppress the expression of fibrosis-associated proteins including COL1, COL3, and smooth muscle actins (Wang et al., 2012). We also found that oral *Z. jujube* decreased the tissue concentration of TGF-β and the subsequent formation of adhesion bands. The TGF-β-induced mRNA upregulation of α-SMA was also significantly diminished by *Z. jujube* treatment.

In the clinical context, the formation of adhesion bands in the uterus presents numerous complications, impacting both maternal health and pregnancy outcomes. Adhesion bands prevent normal endothelial regeneration in the menstrual cycles resulting in infertility (Evans-Hoeker and Young, 2014). Adhesion bands interfere with the blastocyte implantation process and restrain the blood supply of the uterus resulting in a low pregnancy rate and/or unsuccessful pregnancy among individuals affected by IUA (March, 2011). The results obtained in this study demonstrated the protective effect of *Z. jujube* on IUA complications. The implantation success rate and pregnancy rate were significantly enhanced by *Z. jujube* treatment. In addition, the pregnancy outcomes were significantly enhanced by *Z. jujube* as the number of total and live babies increased in pregnant rats and babies had higher weights. Another noteworthy observation was the normal and balanced growth pattern in the babies of *Z. jujube*-treated rats compared to the IUA control group.

Briefly, *Z. jujube* is a plant with potential therapeutic properties that could facilitate endometrial regeneration and alleviate adhesion formation. Several key attributes contribute to its efficacy in this context: First, *Z. jujube* possesses anti-inflammatory properties, which may help reduce inflammation and granuloma tissue formation (Zhu et al., 2024). Inflammation is a critical factor in various diseases and conditions, including endometrial dysfunction. By mitigating inflammation, *Z. jujube* may create a more favorable environment for endometrial regeneration. Additionally, *Z. jujube* has been shown to inhibit the activity of NF-κB, a transcription factor that regulates the production of pro-inflammatory cytokines (Miao et al., 2020). This modulation of NF-κB activity could play a crucial role in reducing inflammation and promoting tissue regeneration (Miao et al., 2020). It has also demonstrated the ability to inhibit the formation of bacterial biofilms, leading to a reduction in the thickness and adhesion of these biofilms. The inhibitory effects on biofilm formation could be beneficial in preventing infection and promoting a healthy environment for endometrial regeneration (Miao et al., 2020). This plant contains various active compounds, such as triterpenic acids, ursolic acid, and oleanolic acid, which are believed to possess anti-inflammatory properties (Yu et al., 2012). These compounds may work synergistically to promote endometrial regeneration and reduce adhesion formation (Kang et al., 2015). Furthermore, *Z. jujube* contains alkaloids, which are known for their antioxidant and antiviral properties (Zare-Zardini et al., 2013). These alkaloids may further contribute to the overall therapeutic effects of *Z. jujube* in the context of endometrial health.

The endometrium is a highly regenerative tissue that undergoes cyclical processes of growth, differentiation, and shedding, regulated by hormones such as estrogen and progesterone (Gargett et al., 2012). The potential therapeutic properties of *Z. jujube*, as discussed above, may provide additional support to the endometrium during these cyclical changes, promoting optimal tissue regeneration and reducing adhesion formation. However, further research is necessary to fully understand the effects of *Z. jujube* on endometrial health and its potential clinical applications.

In conclusion, our findings suggest that *Z. jujube* holds promise as a safe therapeutic candidate for mitigating intra- and extra-uterine adhesion bands. The protective effects of *Z. jujube* in decreasing the formation of adhesion bands may be attributed to its inhibitory effects on inflammation, oxidative stress, and fibrosis. However, further research is warranted to elucidate the exact mechanisms underpinning *Z. jujube*’s protective effects and to assess its efficacy in other animal models and clinical trials.

## Data Availability

The raw data supporting the conclusion of this article will be made available by the authors, without undue reservation.

## Abbreviations

CAT, catalase; H&E, Hematoxylin and Eosin; IL-1β, interleukin-1 beta; IL-6, interleukin-6; IFN-γ, Interferon gamma; MDA, malonyl dialdehyde; SOD, superoxide dismutase; TGF-β, transforming growth factor β; TNF-α, necrosis factor-alpha; α-SMA, α-smooth muscle actin; IUA, Intrauterine adhesion; *Z. jujube*, *Ziziphus jujube*.
